# Supplementary material for: Remdesivir improves biomarkers associated with disease severity in COVID-19 patients treated in an outpatient setting
Source: Commun Med (Lond). 2023 Jan 3;3:2. doi: 10.1038/s43856-022-00232-2 (PMC9809529; doi:10.1038/s43856-022-00232-2)
Supplement: Supplementary file 2 — Supplementary Information [file 43856_2022_232_MOESM2_ESM.pdf]

Remdesivir improves biomarkers associated with disease severity in COVID-19 patients treated in an outpatient setting

David Z. Pan[#], Pamela M. Odorizzi[#], Andre Schoenichen, Mazin Abdelghany, Shuguang Chen, Anu Osinusi, Scott D. Patterson, Bryan Downie, Kavita Juneja, and Jeffrey J. Wallin*

[#]These authors contributed equally to this work.; *Corresponding author

Corresponding author email: jeffrey.wallin@gilead.com

Gilead Sciences Inc., 333 Lakeside Dr., Foster City, CA, 94404

Supplementary Materials

Supplementary Figure 1. Biomarker associations with gender and age in RDV and placebo treatment arms.

Supplementary Figure 2. Baseline Biomarkers Stratified by the Primary Endpoint of the PINETREE study.

Supplementary Figure 3. Changes in inflammation, coagulation, and hematologic biomarkers in RDV- vs. placebo-treated patients.

Supplementary Table 1. Baseline characteristics and demographics of PINETREE study participants who consented to optional biomarker collection

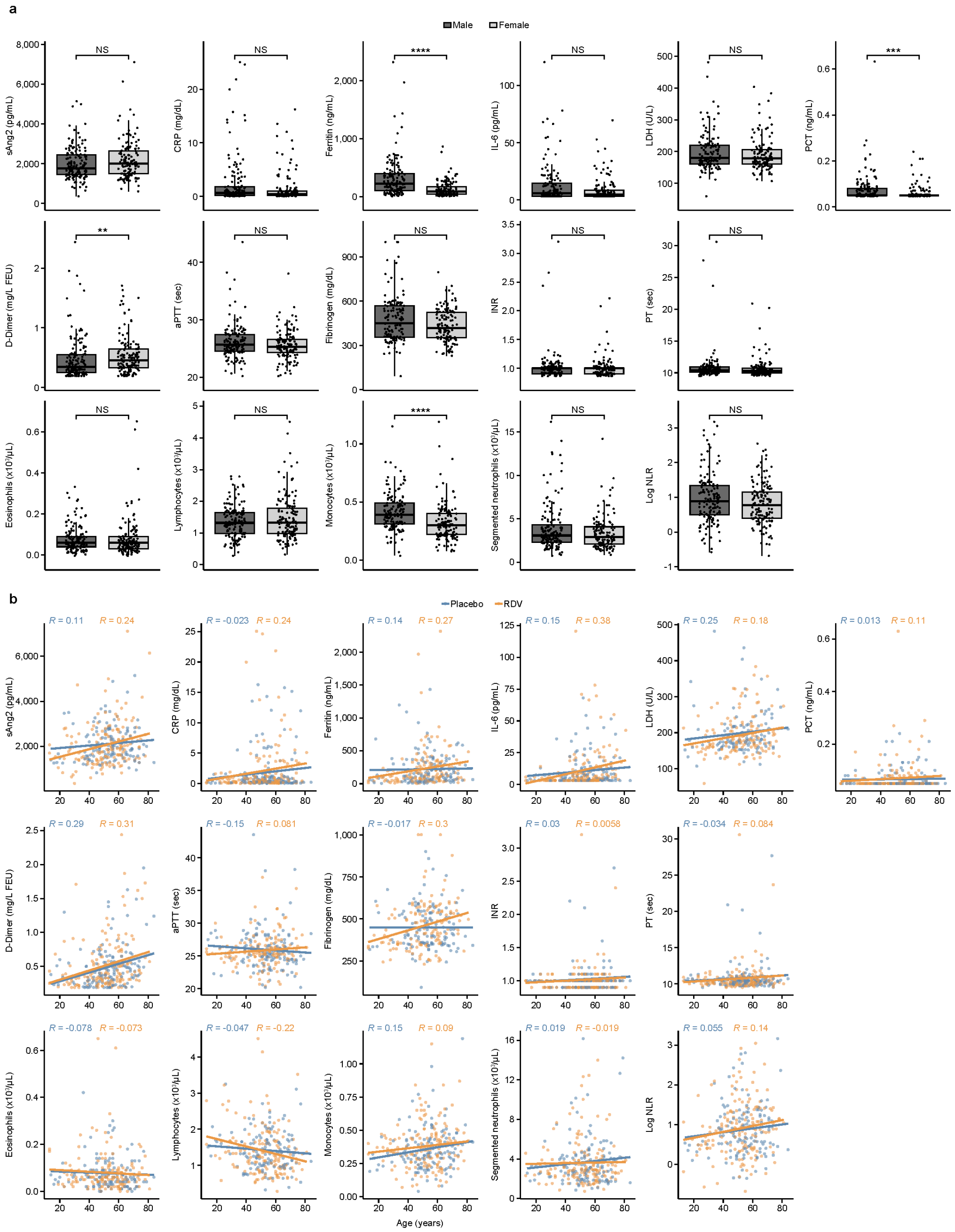

Supplementary Figure 1. Biomarker associations with gender and age in RDV and placebo treatment arms. A) Box plots of biomarkers at baseline (day 1) in males and females. Non-parametric Wilcoxon rank sum test used to determine significance of differences. *FDR<0.05; **FDR<0.01; ***FDR<0.001, ****FDR<0.0001. The line within each box denotes the median and each box extends to the 25th and 75th percentiles. The whiskers indicate 1.5 interquartile range. Significance was determined using a Wilcoxon Rank Sum Test. **B)** Spearman correlation between age and biomarkers at baseline (day 1) stratified by treatment arm. R indicates the Spearman correlation coefficient. (soluble Angiopoietin 2 (sAng2): n=293; C-reactive protein (CRP): n=295; ferritin: n=280; interleukin-6 (IL-6): n=271; lactate dehydrogenase (LDH): n=251; procalcitonin (PCT): n=294; D-Dimer: n=290; activated partial thromboplastin time (aPTT): n=282; Fibrinogen: n=265; international normalized ratio (INR): n=282; prothrombin time: n=282; eosinophils: n=286; lymphocytes: n=286; monocytes: n=286; neutrophils: n=286; neutrophil-to-lymphocyte ratio (NLR): n=286).

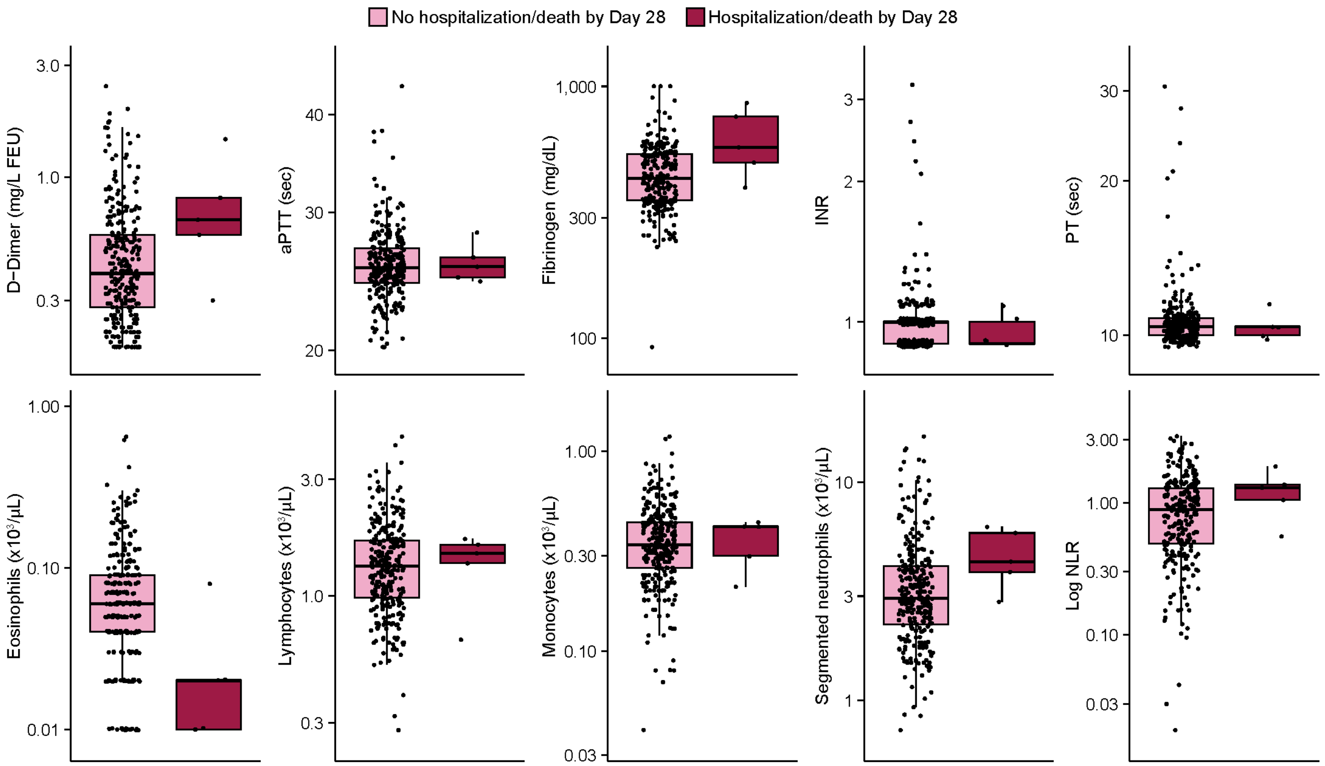

Supplementary Figure 2. Baseline Biomarkers Stratified by the Primary Endpoint of the PINETREE study. Box plots of biomarkers that did not reach statistical significance (FDR>0.05) in patients who met the primary endpoint of hospitalization or death by day 28 (purple) vs. those who did not (pink). The line within each box denotes the median and each box extends to the 25th and 75th percentiles. The whiskers indicate 1.5 interquartile range. (D-Dimer: n=290; activated partial thromboplastin time (aPTT): n=282; Fibrinogen: n=265; international normalized ratio (INR): n=282; prothrombin time: n=282; eosinophils: n=286; lymphocytes: n=286; monocytes: n=286; neutrophils: n=286; neutrophil-to-lymphocyte ratio (NLR): n=286).

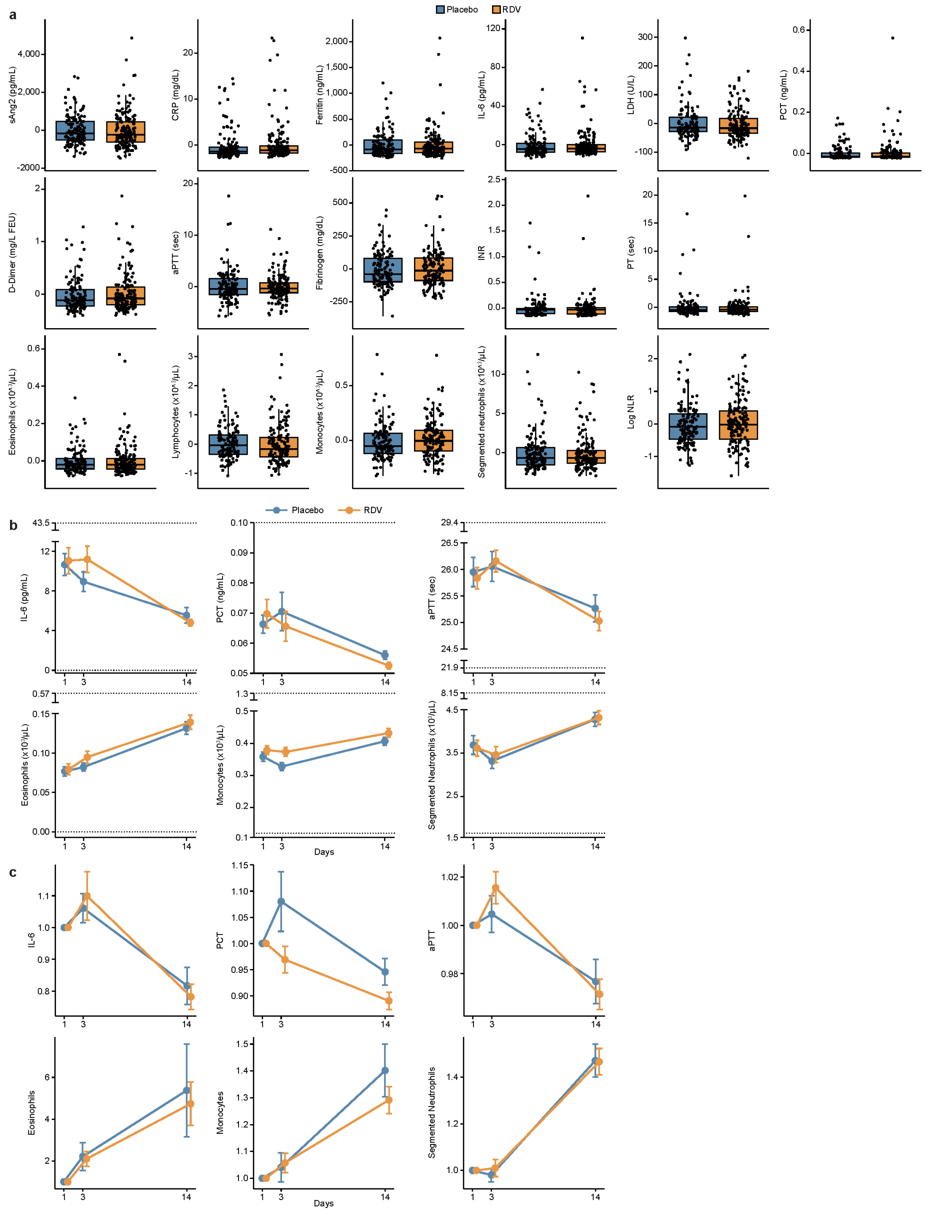

Supplementary Figure 3. Changes in inflammation, coagulation, and hematologic biomarkers in RDV- vs. placebo-treated patients. A) Box plots of baseline levels of biomarkers in RDV- and placebo-treated patients. All comparisons between placebo and RDV were not significant. The line within each box denotes the median and each box extends to the 25th and 75th percentiles. The whiskers indicate 1.5 interquartile range. Significance was determined using a Wilcoxon Rank Sum Test. (soluble Angiopoietin 2 (sAng2): n=293; C-reactive protein (CRP): n=295; ferritin: n=280; interleukin-6 (IL-6): n=271; lactate dehydrogenase (LDH): n=251; procalcitonin (PCT): n=294; D-Dimer: n=290; activated partial thromboplastin time (aPTT): n=282; Fibrinogen: n=265; international normalized ratio (INR): n=282; prothrombin time: n=282; eosinophils: n=286; lymphocytes: n=286; monocytes: n=286; neutrophils: n=286; neutrophil-to-lymphocyte ratio (NLR): n=286). **B)** Longitudinal plots of biomarker absolute value changes (mean with standard error) in RDV- (yellow) vs. placebo-treated (blue) patients. Dashed lines indicate normal ranges or expected values in healthy individuals. **C)** Longitudinal plots of fold change (mean with standard error) in biomarkers in RDV- (yellow) vs placebo-treated (blue) patients. Significance is determined by a linear mixed effects model (LMM). The number of patients used in the LMM for each biomarker is provided in Supplementary Data 1.

Supplementary Table 1. Baseline characteristics and demographics of PINETREE study participants who consented to optional biomarker collection

	RDV<br>n=168	Placebo<br>n=144
Mean age, y (SD)	51 (14)	53 (14)
Aged ≥60 y, n (%)	55 (33)	44 (31)
US region, n (%)	156 (93)	134 (93)
Female sex at birth, n (%)	78 (46)	69 (48)
Race, n (%)		
White	147 (88)	130 (90)
Black	10 (6)	7 (5)
American Indian or Alaska Native	2 (1)	2 (1)
Hispanic or Latinx ethnicity, n (%)	72 (43)	55 (38)
Comorbidities, n (%)		
Diabetes mellitus	109 (65)	91 (63)
Obesity	93 (55)	82 (57)
Hypertension	87 (52)	66 (46)
Chronic lung disease	33 (20)	31 (22)
Current cancer	11 (7)	12 (8)
Cardiovascular or cerebrovascular disease	10 (6)	9 (6)
Immunocompromised	11 (7)	3 (2)
Chronic kidney disease, mild or moderate	4 (2)	10 (7)
Chronic liver disease	0 (0)	0 (0)
Median (IQR) time from PCR confirmation, days	3 (2, 4)	2 (1, 3.5)
Median exposure to study drug, doses received (IQR)	3 (3, 3)	3 (3, 3)

IQR, interquartile range; SD, standard deviation.
