# Supplementary material for: Remdesivir improves biomarkers associated with disease severity in COVID-19 patients treated in an outpatient setting
Source: Commun Med (Lond). 2023 Jan 3;3:2. doi: 10.1038/s43856-022-00232-2 (PMC9809529; doi:10.1038/s43856-022-00232-2)
Supplement: Supplementary file 5 — Reporting Summary [file 43856_2022_232_MOESM5_ESM.pdf]

nature portfolio

Corresponding author(s): Jeffrey Wallin

Last updated by author(s): Dec 13, 2022

# Reporting Summary

Nature Portfolio wishes to improve the reproducibility of the work that we publish. This form provides structure for consistency and transparency in reporting. For further information on Nature Portfolio policies, see our Editorial Policies and the Editorial Policy Checklist.

## Statistics

For all statistical analyses, confirm that the following items are present in the figure legend, table legend, main text, or Methods section.

| n/a | Confirmed | |
|---|---|---|
| ☐ | ☒ | The exact sample size (*n*) for each experimental group/condition, given as a discrete number and unit of measurement |
| ☐ | ☒ | A statement on whether measurements were taken from distinct samples or whether the same sample was measured repeatedly |
| ☐ | ☒ | The statistical test(s) used AND whether they are one- or two-sided<br>*Only common tests should be described solely by name; describe more complex techniques in the Methods section.* |
| ☐ | ☒ | A description of all covariates tested |
| ☐ | ☒ | A description of any assumptions or corrections, such as tests of normality and adjustment for multiple comparisons |
| ☐ | ☒ | A full description of the statistical parameters including central tendency (e.g. means) or other basic estimates (e.g. regression coefficient) AND variation (e.g. standard deviation) or associated estimates of uncertainty (e.g. confidence intervals) |
| ☐ | ☒ | For null hypothesis testing, the test statistic (e.g. *F*, *t*, *r*) with confidence intervals, effect sizes, degrees of freedom and *P* value noted<br>*Give P values as exact values whenever suitable.* |
| ☐ | ☒ | For Bayesian analysis, information on the choice of priors and Markov chain Monte Carlo settings |
| ☐ | ☒ | For hierarchical and complex designs, identification of the appropriate level for tests and full reporting of outcomes |
| ☐ | ☒ | Estimates of effect sizes (e.g. Cohen's *d*, Pearson's *r*), indicating how they were calculated |

*Our web collection on statistics for biologists contains articles on many of the points above.*

## Software and code

Policy information about availability of computer code

| | |
|---|---|
| Data collection | No software was used for data collection. |
| Data analysis | All statistical analyses were performed using R version 4.0.5. Statistical tests were stated in the manuscript and were performed using base R except for linear mixed effects models which used the lmer4 package. |

For manuscripts utilizing custom algorithms or software that are central to the research but not yet described in published literature, software must be made available to editors and reviewers. We strongly encourage code deposition in a community repository (e.g. GitHub). See the Nature Portfolio guidelines for submitting code & software for further information.

## Data

Policy information about availability of data

All manuscripts must include a data availability statement. This statement should provide the following information, where applicable:

- Accession codes, unique identifiers, or web links for publicly available datasets
- A description of any restrictions on data availability
- For clinical datasets or third party data, please ensure that the statement adheres to our policy

Any requests for use of the data in this study must be sent to the corresponding author. The request will be examined with respect to the Informed Consent Form relevant for this clinical trial. Source data for the figures are available in Supplementary Data 1.

## Human research participants

Policy information about studies involving human research participants and Sex and Gender in Research.

| | |
|---|---|
| Reporting on sex and gender | Sex was collected and based on self-reporting. It was used as a covariate in statistical analyses. Overall the study was balanced between males (n=155) and females (n=146) with balanced numbers in the placebo (males n=69, females n=69) and treatment (males n=86, females n=77) arms. |
| Population characteristics | Age was used as a covariate in the statistical analyses. |
| Recruitment | The PINETREE study was a randomized, double-blind, placebo-controlled Phase 3 clinical trial conducted at sixty-four outpatient centers and skilled nursing facilities. Enrollment included participants with a positive SARS-CoV-2 test at high risk for severe COVID-19, including those >/= 60 years or >/= 18 years (or, where permitted by law, 12-17 years weighing >/= 40 kg) and/or those with one or more of the following risk factors: chronic lung disease, hypertension, cardiovascular disease, diabetes, obesity, immunocompromised, chronic kidney disease, chronic liver disease, cancer, or sickle-cell disease. Individuals vaccinated for COVID-19 could not participate in the study. |
| Ethics oversight | The trial was approved by the institutional review board or ethics committee at each site and was conducted in compliance with the Declaration of Helsinki Good Clinical Practice guidelines and local regulations. The names and approval numbers of each IRB/IEC are included in Supplementary Data 1. |

Note that full information on the approval of the study protocol must also be provided in the manuscript.

# Field-specific reporting

Please select the one below that is the best fit for your research. If you are not sure, read the appropriate sections before making your selection.

☒ Life sciences ☐ Behavioural & social sciences ☐ Ecological, evolutionary & environmental sciences

For a reference copy of the document with all sections, see nature.com/documents/nr-reporting-summary-flat.pdf

# Life sciences study design

All studies must disclose on these points even when the disclosure is negative.

| | |
|---|---|
| Sample size | As biomarker collection was optional in this study, we used as many samples as possible with biomarker data. The study included 562 participants who received at least 1 dose of study drug, of which 312 consented for longitudinal biomarker assessments at baseline, day 3, and day 14. Although the sample size of 312 was adequate for most statistical analyses, we have clearly stated in the manuscript analyses where we were limited by sample size available. |
| Data exclusions | Missing data at any time point was excluded from analyses. |
| Replication | As this is a randomized, double-blind, placebo-controlled Phase 3 clinical trial specifically about the relationship of remdesivir treatment and relevant biomarkers, there is not currently another dataset that could used to replicate these results. |
| Randomization | Patients were randomized to the placebo and treatment arms. |
| Blinding | This study was double-blinded. |

# Reporting for specific materials, systems and methods

We require information from authors about some types of materials, experimental systems and methods used in many studies. Here, indicate whether each material, system or method listed is relevant to your study. If you are not sure if a list item applies to your research, read the appropriate section before selecting a response.

## Materials & experimental systems

| n/a | Involved in the study | |
|---|---|---|
| ☒ | ☐ | Antibodies |
| ☒ | ☐ | Eukaryotic cell lines |
| ☒ | ☐ | Palaeontology and archaeology |
| ☒ | ☐ | Animals and other organisms |
| ☐ | ☒ | Clinical data |
| ☒ | ☐ | Dual use research of concern |

## Methods

| n/a | Involved in the study | |
|---|---|---|
| ☒ | ☐ | ChIP-seq |
| ☒ | ☐ | Flow cytometry |
| ☒ | ☐ | MRI-based neuroimaging |

# Clinical data

Policy information about clinical studies

All manuscripts should comply with the ICMJE guidelines for publication of clinical research and a completed CONSORT checklist must be included with all submissions.

| | |
|---|---|
| Clinical trial registration | NCT04501952 |
| Study protocol | This trial was conducted according to protocol without substantial deviations and registered with ClinicalTrials.gov (NCT04501952) |
| Data collection | Collection of data occurred between September 18, 2020 and May 6, 2021 across sixty-four outpatient centers and skilled nursing facilities. |
| Outcomes | The primary outcome measures relevant to this manuscript were as follows:<br>1. Percentage of Participants With Coronavirus Disease 2019 (COVID-19) Related Hospitalization (Defined as at Least 24 Hours of Acute Care) or All-Cause Death by Day 28 [ Time Frame: Randomization up to Day 28 ]. The composite outcome of COVID-19 related hospitalization (defined as at least 24 hours of acute care) or all-cause death by Day 28 was derived by combining the available all-cause death and COVID-19 related hospitalization reported by the site. The first COVID-19 related hospitalization was used for the percentage of COVID-19 related hospitalization or all-cause death. The percentage of the composite outcome was from the Kaplan-Meier estimate. |
